# Providing a Helping Hand: Metabolic Regulation of T Follicular Helper Cells and Their Association With Disease

**DOI:** 10.3389/fimmu.2022.864949

**Published:** 2022-04-14

**Authors:** Colleen L. Mayberry, Natalie A. Logan, John J. Wilson, Chih-Hao Chang

**Affiliations:** ^1^ The Jackson Laboratory, Bar Harbor, ME, United States; ^2^ Graduate School of Biomedical Sciences and Engineering, University of Maine, Orono, ME, United States; ^3^ Graduate School of Biomedical Sciences, Tufts University School of Medicine, Boston, MA, United States

**Keywords:** T follicular helper cells, Tfh differentiation, cellular metabolism, germinal center, infection

## Abstract

T follicular helper (Tfh) cells provide support to B cells upon arrival in the germinal center, and thus are critical for the generation of a robust adaptive immune response. Tfh express specific transcription factors and cellular receptors including Bcl6, CXCR5, PD-1, and ICOS, which are critical for homing and overall function. Generally, the induction of an immune response is tightly regulated. However, deviation during this process can result in harmful autoimmunity or the inability to successfully clear pathogens. Recently, it has been shown that Tfh differentiation, activation, and proliferation may be linked with the cellular metabolic state. In this review we will highlight recent discoveries in Tfh differentiation and explore how these cells contribute to functional immunity in disease, including autoimmune-related disorders, cancer, and of particular emphasis, during infection.

## Introduction

T follicular helper cells (Tfh) are a critical cellular subset in adaptive immunity, serving as a bridge between both cell-mediated and humoral immune responses. Both Tfh and follicular regulatory T (Tfr) cells are responsible for modulating a germinal center (GC) response, an antibody response essential for humoral immunity in response to antigen (1–4). Tfr cells express CD25 and transcription factor (TF) Foxp3, and can secrete anti-inflammatory cytokines, such as TGF-β (5). Tfh, on the other hand, express CXCR5, ICOS, TF Bcl6, and produce IL-21 and IL-4 (6–8). Induction of TF Bcl6 in these cells is largely due to IL-6 presence during differentiation (9, 10). In addition to Bcl6, these cells also express signal transducers and transcriptional activators (STAT) 1, 3, and 4, Batf, Maf, and Acl2 (11). The cytokines that are present during peripheral T cell differentiation (at the time of antigen encounter) can dictate which cell subtype the naïve CD4^+^ T cells will differentiate into, largely through regulation of TF expression (12). The cocktail of TFs present in a cell controls the surface expression of specific receptors. For Tfh, the surface markers expressed are CXCR5, ICOS, CD40L, PD-1, and BTLA (7, 13). It is the differential expression of these receptors, in addition to the intracellular expression of TFs, that are used widely to define the Tfh subset. Although TF Bcl6 is considered the master regulator of Tfh, these cells also express cytoplasmic signaling molecule SLAM-associated protein (SAP), which is involved in the secretion of IL-21 and terminal Tfh differentiation (7, 14).

The mechanisms underlying Tfh differentiation are complex; This review summarizes the key findings in the processes underlying Tfh differentiation with a particular emphasis on the metabolic pathways involved, and the mechanisms by which Tfh may influence the pathogenesis of infection, cancer, and autoimmune diseases. The insights provided from the work cited herein are key for the development of improved, targeted, therapeutics.

## Differentiation of Tfh

**Figure 1:**
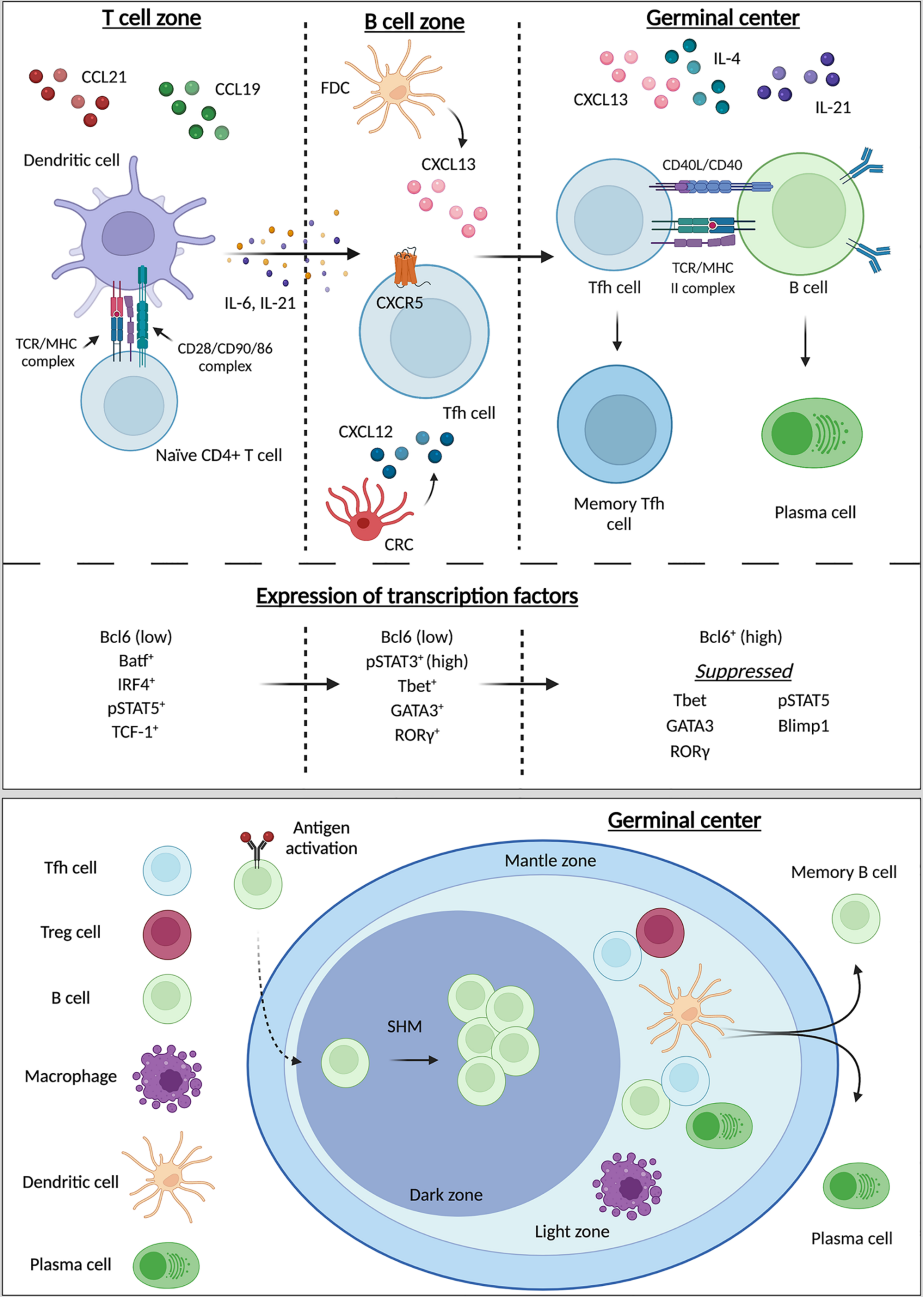
Differentiation, maturation of Tfh and germinal center organization. Tfh differentiation progressively occurs in three different zones: the T cell zone, the B cell zone, and the germinal center (GC). From the T cell zone to the GC, naïve CD4^+^ T cells differentiate into Tfh with final maturation occurring within the GC. In the T cell zone interactions between CD4^+^ naïve T cells and dendritic cells (DC) initiate Tfh differentiation and promote the release of IL-6, IL-21, and the upregulation of CXCR5 on the pre-Tfh. These pro-Tfh cytokines result in the release of CXCL13 by follicular dendric cells (FDCs) and CXCL12 by CXCL12-producing reticular cells (CRCs) in the B cell zone. Recognition of CXCR5 by CXCL13 results in recruitment of the immature Tfh into the B cell zone. Both CXCR5 and ICOS signaling promote homing of the now-mature Tfh into the GC. Interactions with B cells in the GC result in the maturation of B cells into plasma cells for the production of antibodies. During these three stages of Tfh differentiation the transcriptional environment changes as differentiation progresses. Initially expression Bcl6 is low, and the pre-Tfh cell enhances expression of Batf, IRF1, pSTAT5, and TCF-1. During the second stage of development Bcl6 expression remains low while pSTAT3, Tbet, and RORg increase in expression. In the final stage of Tfh maturation Bcl6 is now highly expressed, while suppression of Tbet, GATA3, RORg, pSTAT5, and Blimp1 are suppressed. The GC is comprised of three zones: the mantle zone, light zone, and dark zone. The light zone primarily houses T cells and the majority of T:B cell interactions occur here. Within the dark zone B cells undergo somatic hyper mutation (SHM) for differentiation into plasma cells and long-lived memory B cells. Created with BioRender.com.

It has been proposed that Tfh differentiation progressively occurs in three different steps (7, 15–18) (**Figure 1**). The localization of Tfh may contribute to overall function, resulting in subdifferentiation of tissue-specific Tfh lineages. The initial phase in Tfh differentiation occurs during the localization of progenitor Tfh to B cell-containing follicles (10). The trigger for this migration event is through antigen signaling by dendritic cells resulting in T cell receptor (TCR) activation on naïve CD4^+^ T cells (19). During the interaction of antigen-presenting cells, such as dendritic cells (DC) and CD4^+^ T cell priming event, two interactions underly initial signaling necessary for pre-Tfh commitment, first, through binding of the TCR to the major histocompatibility complex (MHC), and secondly, binding of CD28 on T cells by DC-expressing CD80/86 in a co-stimulation mechanism. Interactions between naïve T cells and DCs are further regulated by the expression of cytokines and receptors including IL-6, ICOS, and IL-2 (19, 20). Secretion of IL-6, largely by DCs, stimulates the production of transcription factors central to Tfh differentiation, primarily Bcl6, but also includes TCF-1 and LEF-1, among others (21, 22). The nuances of DC modulation of Tfh differentiation have been extensively reviewed (23, 24). Induction of Bcl6 signaling results in expression CXCR5 on the cell surface, regulates Tfh intracellular calcium signaling, and modulates expression of CD40L (9, 10, 25). While the deletion of one copy of the Bcl6 gene did not significantly alter activation of Tfh or localization of Tfh to follicles, GC formation and maintenance was inhibited. Moreover, overexpression of CD40L could partially rescue GC formation and Tfh maintenance after deletion of the single copy of the Bcl6 gene (25). This highlights the importance of Bcl6 for Tfh maintenance and signifies that there may be overlapping mechanisms of Bcl6 and CD40L in mediating T:B cell interactions. Co-stimulatory receptor OX40 also regulates Tfh differentiation, though is attributed as both a positive and a negative regulator: LCMV-infected OX40-deficient mice exhibit impaired Tfh differentiation (26) while enhanced OX40 signaling, in combination with anti-PD-1 blockade, in *Plasmodium* infection diminished the Tfh response, through upregulation of inhibitory Blimp1 and IFN-γ signaling (27). ICOS expression also plays a major role in the regulation of Tfh migration and differentiation (28, 29). Likewise, its corresponding ligand ICOSL, expressed by B cells, is important for Tfh differentiation, through manipulation of the glycolytic environment of Th cells (24, 30). Thus, expression of cellular receptors can largely influence the cellular differentiation fate.

Fibroblastic reticular cells produce chemoattractants CCL19, CCL21, and CXCL12; expression of CCL19/21 induces migration of CCR7-expressing naïve CD4^+^ T cells and DCs to the T cell zone, resulting in T cell priming (7, 31–34). Then, a series of changes occur. CXCL13, responsible for recognition of CXCR5, is produced by follicular dendritic cells (FDCs), one of two stromal cell types contained within B cell follicles, with the other being CXCL12-producing reticular cells (CRCs), responsible for recognition of CXCR4. The production of CXCL13/12 increases migration of B cells during the induction of a germinal center response (35, 36). Interactions between DCs and naïve CD4^+^ T cells in the T cell zone promote CD4^+^ Tfh differentiation resulting in the upregulation of CXCR5 and subsequent down regulation of CCR7, and the migration of the T cells to the CXCL13-rich B cell zone contained within follicles (23).

Although CXCR5 expression is sufficient for T cell arrival at the B cell zone, not all CXCR5-expressing T cells cross the T-B cell junction. Arrival within the B cell zone is highly dependent on PI3K signaling (37). Expression of CXCR5 and ICOS on the cell surface promotes PI3K signaling, while T cell PD-1 interaction with B cell PD-L1 results in PI3K inhibition; inhibition which is overcome by the ICOS-linked PI3K activation (38). In addition to increasing PI3K activation, ICOS also enhances the formation of T cell pseudopodia and migration (39). Lastly, T cells from the T cell zone, migrate across the T-B cell junction, marking phase two of Tfh differentiation, into the B cell zone (7). Signaling pathways necessary for early Tfh differentiation are controlled by CXCR5 expression and Bcl6, as well as pathways largely regulated by DCs, including IL-6, ICOS, TCR, and IL-2 signaling pathways (20). Some of these interleukins are also usurped during disease. For example, *Plasmodium* infection increases IL-6, resulting in enhanced B- and CD4^+^ T cell activation (40). This in turn promotes parasite-specific antibody production. Together, this demonstrates the critical roles that signaling networks may play early in the fate of Tfh.

Subsequently following T:B cell interactions, the third phase of Tfh differentiation occurs. This final phase in the maturation of Tfh happens in specialized structures within B cell zones known as germinal centers (GCs) (41, 42). Tfh express SAP resulting in secretion of IL-21, which is not only necessary for final maturation of Tfh within the GC but is also critical for normal B cell development (43, 44). Within GCs, B cells produce high-affinity antibodies and further differentiate into memory B cells or plasma cells after undergoing randomized somatic hypermutation (SHM) which generates an improved antigen affinity (45). Moreover, GCs are divided into two distinct compartments, the light zone (LZ) and dark zone (DZ), each compartment caters to specific GC-associated cell types. The light zone primarily houses Tfh and Tfr cells that have entered the GC. Here, Tfh assist in the production of GCs and aid B cells, providing signaling for B cell differentiation into plasmablasts and plasma cells for production and secretion of antibodies. Meanwhile, FoxP3^+^ Tfr are also critical for GC regulation, as these cells suppress Tfh/B cell signaling to prevent excessive antibody production (46). Tfr are a specialized subset of Treg cells that have adopted a Tfh phenotype, likely due to the cytokine milieu in which they were differentiated. This generates a Treg cell type that is capable of homing to GC, due to high expression of Tfh markers CXCR5 and Bcl6 (47). The numerical balance between Tfh and Tfr is a large factor in regulation of the GC microenvironment and is essential for avoiding the development of autoimmunity. Meanwhile, the DZ supports B cell SHM and B cell differentiation and maturation (45). Underlying B cell SHM is the expression of CXCR4. Expression of CXCR4 retains B cells within the DZ where these cells undergo rapid proliferation until CXCR4 expression decreases, at which time these cells migrate into the LZ where interactions with FDCs and Tfh occur (48) (**Figure 1**).

Although Tfh reach maturation within GCs, Tfh can migrate between GCs or even through blood or lymph (49). During this migration Bcl6 is downregulated, and these Tfh are now termed memory Tfh, and upon IL-7Rα upregulation, become resting memory Tfh. Resting memory Tfh express even lower levels of Bcl6. Memory Tfh are found in the blood, lymph, and secondary lymphoid organs. It is estimated that approximately 20% of the circulating CD4^+^ memory T cells are memory Tfh (50). These memory Tfh express comparable levels of CXCR5 to Tfh which reside within lymph nodes, though they express lower levels of PD-1 and ICOS (51). It is speculated that circulating Tfh may differentiate into memory Tfh. Memory Tfh may circulate scouring for antigen. Upon arrival within lymph nodes, these cells require priming by DCs to functionally produce and secrete cytokines, which are produced at higher levels than lymph node-resident Tfh. It is posited that circulating memory Tfh are on the frontline for defense against re-exposure to antigen (50).

## Cytokine and Transcriptional Regulation of Tfh Differentiation

The transcriptional environment of cells can promote Tfh differentiation (52). During cell division and growth, differential expression of transcription factors (TFs) influences the maturation and differentiation of those cells. Multiple TFs have been positively and negatively associated with Tfh differentiation (52) (**Figure 1**).

Bcl6 is a transcriptional repressor critical for Tfh differentiation, as these cells cannot form in its absence (53, 54). Moreover, induction of Bcl6 induces expression of CXCR5, ICOS, and PD-1 and robust Tfh differentiation in *Bcl6^-/-^* mice (54–56). It has been proposed that Bcl6 dictates Tfh differentiation through both direct and indirect methods. *Via* direct means, Bcl6 can modulate T cell fate through DNA-binding capabilities; Bcl6 represses signaling pathways that would generate alterative CD4^+^ T cell subtypes (56). Through indirect means, acting as a ‘repressor of repressors’ Bcl6 can inhibit miRNAs that would otherwise serve to inhibit Tfh differentiation, typically through inhibition of a Tfh-specific marker like CXCR5 (56). Bcl6 can also indirectly enhance CXCR5 and PD-1 signaling through suppression of p-selectin glycoprotein 1 (PDG-1) (57) and through suppression of Blimp1 (58). Although Bcl6 suppression of alternative TFs has been reported, including T-bet, ROR-γ, and GATA-3 (59, 60), direct modulation of these TFs that are often expressed concurrently with Bcl6 require further experimentation. The differential TF expression may generate specific genetic patterns under varying conditions. Bcl6 can also influence the cytokine milieu during differentiation (8) and receptor expression and cellular migration (8, 61, 62). Thus, while the many facets of its involvement in direct and indirect manipulation of T cell lineages are still unraveling, Bcl6 is clearly essential for Tfh differentiation.

STATs promote the differentiation of Tfh, with particular emphasis on STATs 1, 3, and 4 (11, 63). Expression of CXCR5 and Bcl6 enhances activity of STAT1 in murine cells to interact with Maf and Batf, which in turn further enhance CXCR5 expression (52, 64). Transcription factor Maf is very important for induction of Tfh. Maf expression enhances CXCR5, IL-21, and IL-4, with Maf directly influencing the production of IL-17 and IL-21, all of which are critical for Tfh differentiation and function (28, 65). Maf is activated through ICOS signaling (65). Batf enhances Bcl6 expression (66, 67). Under conditions of low Batf expression *in vivo*, induction of both Bcl6 and Maf is necessary for the induction of CXCR5 (66). Although expression of Batf is not largely enhanced in Tfh, mice that fail to express Batf (*Batf* ^-/-^) are incapable of producing Tfh, underscoring the influence of small changes in Batf expression on Tfh differentiation (66).

STAT3 has also been linked with the promotion of Tfh in mouse cells, while STAT3 and STAT4 are important for Tfh induction in humans (52). Although STATs largely enhance Tfh expression through transcriptional regulation, STAT5 negatively influences Tfh differentiation (67, 68). Multiple TFs can bind to the Bcl6 promotor, thereby influencing Bcl6 mRNA expression (69). The binding of TFs to the Bcl6 promotor is influenced by IL-2 expression; under conditions of low IL-2, STAT3 competitively binds to the promotor, enhancing Bcl6 mRNA expression. However, when IL-2 is high, STAT5 instead binds to the Bcl6 promotor, thereby inhibiting Bcl6 expression (69). However, mature GC-Tfh produce high levels of IL-2. It has since been determined that Tfh within GCs maintain IL-2 hyporesponsiveness through IL-6 signaling. IL-6 inhibited the upregulation of CD122 on the cell surface, which would typically result in increased STAT5 signaling and would be thus susceptible to the effects of IL-2 (70).

The negative influence of TFs in Tfh induction has also been described. Expression of Foxp1 and Foxo1 is necessary for maintaining immature CD4^+^ T cells (71), and inhibition of these TFs enhances the induction of Tfh. As such, the regulation of Foxp1 and Foxo1 by other factors directly influences the fate of the immature CD4^+^ T cells. Regulation of ICOS *via* ICOSL stimulated PI3K signaling, downregulates Foxo1, and upregulated Bcl6 signaling (72, 73). Blimp1 is also an important transcriptional regulator that negatively regulates Tfh differentiation (14). Bcl6 and Blimp1 are transcription factors that are antagonistic, known as the Bcl6/Blimp1 axis (74). CD4^+^ T cells that differentiate into non-Tfh lineages express high levels of Blimp1. Moreover, CD4^+^ T cells that fail to express Blimp1 (*Prdm1* ^-/-^) preferentially differentiate into Tfh, at an accelerated rate, *in vivo*, generally resulting in autoimmunity (75).

Together, while this only presents a subset of the TFs involved, the transcriptional profile of cells greatly influences the fate of CD4^+^ T cells during differentiation. The intricacies of TF-Tfh regulation have been thoroughly summarized elsewhere (52, 76, 77). This suggests that CD4^+^ T cell Tfh lineage requires the appropriate balance of the aforementioned factors, which under healthy conditions, are tightly regulated.

Cytokines play a critical role in regulating Tfh differentiation. It is argued that IL-6 is the most important cytokine in the differentiation of Tfh. IL-6 expression initiates Bcl6 induction during pre-Tfh development, while also inducing IL-21 expression in murine CD4 T cells (20). IL-6 can be secreted by a number of immune-related cell types, including DCs, B cells, and macrophages, among others (24). These cells produce IL-6 in response to internal and external pathogen-associated molecular patterns (PAMPs) and damage-associated molecular patterns (DAMPs). While IL-6 is critical for early induction of pre-Tfh phenotype, addition of IL-21 and IL-27 may compensate for the lack of IL-6 in early Tfh differentiation (20, 78–80).

It has long been reported that IL-2 negatively regulates differentiation of Tfh (81). This has been supported by evidence that IL-2/STAT5 signaling impairs expression of Bcl6 (82). This inhibition is through the promotion of Blimp1 signaling, tipping the Bcl6/Blimp1 axis. Moreover, high expression of PD-1 within GCs by Tfh results in the inhibition of IL-2; expression of IL-2 would typically serve to inhibit Tfh differentiation, suggesting that expression of IL-2 may directly affect PD-1 expression, thereby influencing Tfh differentiation fate (82). Interestingly, IL-2 is initially highly expressed during Tfh differentiation, and is necessary for their selective differentiation, and appears to wane as differentiation progresses (81). A complete picture of how differential and temporal IL-2 expression regulates Tfh differentiation requires further experimentation. Though not discussed here, numerous other cytokines can influence Tfh differentiation. Comprehensive reviews of cytokine regulation of Tfh differentiation are available (10, 83, 84).

Until the last decade it was believed that T cells that provided help to B cells were specifically derived from naïve CD4^+^ T cells and were classified as Tfh. However, it is now appreciated that CD8^+^ T cells may, under certain conditions, provide help to B cells in a similar manner, in what is described as Tfh-like activity. One group reported that CD8^+^ T cells could function in this capacity using a mouse model that was deficient in IL-2 (85). These mice generated spontaneous autoimmune disease in what, at the time, was believed to be due to unimpeded CD4^+^ Tfh differentiation. This was supported by the finding that depletion of CD4^+^ T cells in these mice prevented the onset of the autoimmune disease and thus improved survival. However, when the research group depleted CD8^+^ T cells using this model, they found that depletion of CD8^+^ T cells also delayed disease and prolonged survival. RNA sequencing of CD8^+^ T cells in these mice revealed cells that exhibited many of the major Tfh markers, including expression of CXCR5, Bcl6, ICOS, PD-1, and IL-21, and thus may exhibit similar metabolic profiles. These cells, defined as CD8^+^ Tfh-like cell population, are capable of localizing with and providing help to B cells within GCs and appear to contribute to the onset of autoimmune disease, through the dysregulation of Tfh populations (58, 85–89). CD8^+^ Tfh-like cells have been increasingly implicated in immune activity. In patients with rheumatoid synovitis, CD8^+^ Tfh-like cells that express CD40L, and therefore interact with B cells within the GC, have been heavily implicated in disease development (87). These cells also develop under conditions of other inflammatory diseases and in chronic infection and have been described to also express GC-homing Tfh marker CXCR5 (58, 88, 90–92). Moreover, these CD8^+^ Tfh-like cells were capable of supporting survival of B cells *ex vivo* (93). Thus, in certain conditions of disease, CD8^+^ T cells may adopt Tfh-like characteristics and functionality, and therefore these cells cannot be ignored in discussions pertaining to the roles of Tfh in modulation of diseases involving inflammation or infection. Moreover, the metabolic requirements of CD8^+^ Tfh-like cells is an entirely unexplored field of research. Given the implications of these cells that are now emerging for numerous disease states, and given their similarities with canonical Tfh, an understanding of how these cells contribute to the immunological environment is warranted.

Collectively, the local environment directly influences an immune response through regulation of the balance of Tfh induction, through expression of cellular receptors, cytokines, and induction or suppression of transcription factors. Moreover, the intricacies of CD4^+^- versus CD8^+^-derived T cells convolute this multifaceted process. Arguably, metabolic changes underly Tfh differentiation, and these alterations correlate with the expression of Tfh-specific markers.

## Metabolic Regulation of Tfh Differentiation

**Figure 2:**
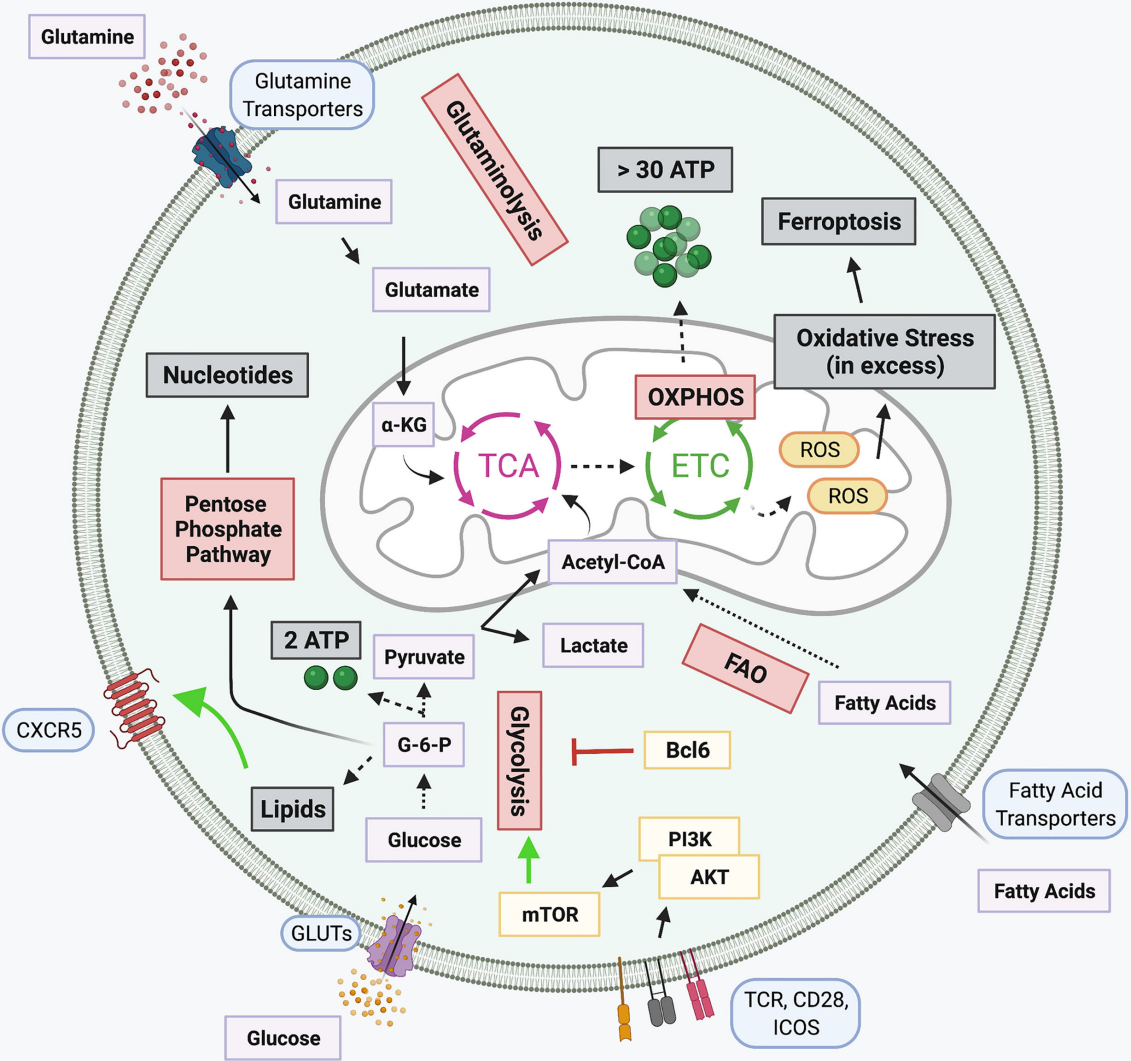
Metabolic pathways in T cells and Tfh-specific alterations. Several signaling networks underly cellular metabolism. Glucose is broken down to produce glycolysis and two ATP molecules in a process known as Glycolysis. Glycolytic intermediates support the pentose phosphate pathway for generation of nucleotides. The pyruvate produced during glycolysis is either converted into lactate or Acetyl-CoA, the latter is subsequently funneled into the tricarboxylic acid (TCA) cycle. Glutaminolysis also fuels the TCA cycle through the breakdown of glutamine into α-ketoglutarate (α-KG). Products of the TCA cycle, along with the oxidation of fatty acids (FAO), support the electron transport chain (ETC) which is coupled with OXPHOS for the generation of over 30 ATP molecules. When in excess, Reactive oxygen species (ROS) generated by the ETC, induce cellular oxidative stress, promoting apoptotic signaling. Several Tfh signaling pathways either promote or inhibit select metabolic pathways. Lipid metabolism, responsible for the generation of lipids, promotes expression of CXCR5 on the cell surface. Bcl6 can repress the transcription of several glycolytic intermediates, thought to prevent differentiation of alternative T cell subtypes, while TCR, CD28, and ICOS signaling in Tfh potently activate glycolysis through PI3K-linked activation of mTOR signaling. Created with BioRender.com.

The metabolic environment has been reported to greatly influence the differentiation of CD4^+^ T cells into functionally mature T cells. Some studies suggest that oxidative phosphorylation (OXPHOS) plays a critical role in Tfh differentiation. However, other sources suggest that glycolysis, rather than OXPHOS is necessary for Tfh induction, due to heightened mTOR and HIF1α signaling (**Figure 2**).

Glycolysis is the process by which glucose is anabolized to produce pyruvate. Glycolysis also involves the production of several intermediates which can be utilized by alternative metabolic pathways, including the pentose phosphate pathway. In addition to the production of pyruvate, glycolysis also generates a small amount of cellular energy. From glycolysis, pyruvate is either transformed into lactate or is shuttled into the mitochondria and fed into the tricarboxylic acid (TCA) cycle. The TCA cycle supports the electron transport chain (ETC). The generated ETC gradient produces significantly more cellular energy than glycolysis in a process known as OXPHOS. However, pyruvate is not the only intermediate fed into the TCA cycle. Products like α-ketoglutarate from glutaminolysis, the breakdown of glutamine, and fatty acids from fatty acid oxidation (FAO) can also be oxidized in the TCA cycle. Acetyl-CoA generated from oxidizing these organic fuel molecules participates in the production of the intra-mitochondrial ETC proton gradient for OXPHOS. Shortcomings of the ETC results in the production of reactive oxygen species (ROS), which when in excess can be detrimental for cellular survival (94–97).

Mitochondrial respiration has been implicated with Tfh cell differentiation. Murine Tfh cells have been reported to exhibit features of ferroptosis, a type of programmed cell death, associated with iron-dependent excessive production of ROS, linked with TCR, CD28, and PD-1 stimulation (98). Moreover, it has been determined that prolonged Tfh-GCB interaction promotes the production of ROS in Tfh, ultimately resulting in cell death *via* ferroptosis (98). As ROS is resultant of ETC and lipid metabolism, this implicates these pathways, and in general, mitochondrial respiration, in the critical regulation of Tfh (98). Several reports highlight the importance of OXPHOS rather than glycolysis in metabolic regulation of Tfh differentiation. This is in part due to a report that Tfh express reduced levels of glycolysis, lower mTOR activity, and decreased mitochondrial respiration (99). At rest, T cells primarily utilize mitochondrial OXPHOS to generate fuel. Following antigen presentation, TCR, CD28, and PI3K/Akt signaling positively favor glycolysis as the primary metabolic pathway. Under these glycolytic conditions, CD4^+^ T cell differentiate into specialized T cell lineages, including Th1 and Th17 cells. However, a MAPK phosphatase, DUSP6, reduces Tfh differentiation through TCR-mediated glycolysis (100). Expression of the Tfh hallmark transcription factor Bcl6 can be modulated through regulation of metabolism. Under glucose restricted conditions or treatment with a potent glycolysis inhibitor 2-deoxyglucose (2-DG), Bcl6 expression is enhanced (101). However, a caveat to this increased Bcl6 expression is that under conditions of low glucose, cellular activation is dampened. When glucose levels are low AMPK is activated, and inhibition of AMPK reduces Bcl6 levels under glucose restriction. However, this Bcl6 induction is insensitive to IL-2 levels. Importantly, inhibition of cellular metabolism promotes Tfh differentiation, prior to cellular maturation, rather than being attributed as a secondary effect. It is thought that the two signaling mechanisms, Bcl6 induction through AMPK signaling under conditions of high cellular proliferation, low energy, and glycolytic demands occur simultaneously (101–103). While this pathway appears to be tightly regulated in this process, the nuances of the mechanisms involved require further clarification.

Other studies have demonstrated that Bcl6 expression results in the suppression of key glycolytic genes (104). Bcl6 can localize directly to foci for glycolytic genes, including Glut1, Glut3, hexokinase 2, and pyruvate kinase M, and functionally repress their transcription in both B and T cells (104). Moreover, this gene repression was sensitive to the expression of IL-2 and CD4^+^ T cells cultured *in vitro* in the absence of glucose exhibited enhanced Tfh differentiation (104). This may provide support for metabolic pathways alternative to glycolysis that drive Tfh differentiation. Primary human GC Tfh sorted from tonsillar tissue analyzed *via* RNA sequencing identified that genes associated with fatty acid oxidation (FAO) were enriched while there was reduced expression of glycolytic genes (105). Moreover, a Seahorse mito-stress test reported higher OXPHOS in GC Tfh cells when compared to other CD4^+^ cell types as the basal oxygen consumption rate, spare respiratory capacity, and ATP production were elevated (105). It is speculated that a precise Bcl6 expression gradient is needed to drive Tfh differentiation from other cell types. This is in conjuncture with a report that T-bet can suppress Bcl6 glycolytic repression to promote glycolytic pathways and Effector T cell function (104). PD-1, highly expressed upon mature Tfh commitment, can also alter metabolic pathways in T cells. PD-1 interaction with ligand PD-L1 suppresses glycolysis and activates genes affiliated with FAO (106). While there is support for OXPHOS for Tfh differentiation and function, reliance on glycolysis is also reported. In a murine study, deletion of an OXPHOS regulator, cytochrome c oxidase, impaired GC formation and antibody responses, though did not impact the overall number of Tfh cells (107), suggesting either their ability to adapt to metabolic changes with ease or the involvement of concurrent metabolic pathways in Tfh regulation.

Intraperitoneal injection of mice with 2-DG results in a hindered immune response through decreased differentiation and impaired function of Tfh (108), establishing a link between glycolysis and proper Tfh function. Glycolysis results in the activation of mTOR-related signaling, through activation of mTOR complex 1 (mTORC1) and complex 2 (mTORC2). Both mTORC1 and mTORC2 have been affiliated with the differentiation of various T cell lineages (109, 110). For Tfh, both mTORC1 and mTORC2 signaling has been reported. It has been shown that *Rptor* and *Rictor* knockout mice limit the humoral immune response through a decrease in the generation of Tfh (110). Raptor and Rictor are critical components of mTORC1 and mTORC2 signaling complexes, respectively. mTORC1 activity has also been linked with enhanced Bcl6 expression, through the downstream activation of 4E-BP1 and eIF4E, *in vitro* (111). Insufficient mTOR activity has been associated with a diminished Tfh response to vaccination (112). In this report, low leptin levels in serum correlated with a diminished Tfh response to vaccination. Leptin is a metabolic hormone secreted by adipose tissue that can modulate cell types of both the innate and adaptive immune systems (113–115). Importantly, the leptin-associated regulation was found to be linked with the stimulation of cellular glycolysis, through STAT3 and mTORC1 signaling (112), both of which are attributed to Tfh differentiation and function. Another group has linked mTOR signaling with the promotion of Tfh differentiation *in vivo*, through ICOS-mediated upregulation of glycolysis (18). However, others suggest that mTORC1 negatively regulates Tfh differentiation through the promotion of IL-2, as determined through *in vitro* methods (99). Although Bcl6 has been attributed to the inhibition of glycolysis, this may be a means of inhibiting differentiation of alternative T cell subtypes, like Th1 cells, thereby indirectly enhancing Tfh differentiation through repressor of repressor functions (104). Furthermore, while this metabolic dichotomy exists, it is important to note that the processes by which cells are differentiated and systemic effector fluctuations, can greatly alter the intra-cellular metabolic environment, thus contributing to the phenotypic and functional profiles of these cells.

There is further support for Tfh dependence on mTOR-mediated glycolysis for proper cellular differentiation and maturation. Cells that exhibit lower levels of mTOR inhibitor PTEN undergo heightened Tfh induction and result in enhanced production of GCs (110, 116). Roquin, an inhibitor of PI3K-mTOR signaling, impairs the Tfh response; knockout of Roquin results in an increase in Tfr (117). As PI3K is intimately linked with Bcl6 induction through ICOS/ICOSL signaling (73), enhanced Tfr differentiation is expected. Furthermore, in Peyer’s patches, mTORC1 and mTORC2 signaling enhances transcriptional promotion of Tfh through ICOS signaling (110). However, mTORC1 signaling has been affiliated with the differentiation of circulating Tfr, which, like Tfh, express CXCR5 and Bcl6. Because of this similarity, it is speculated that mTORC1 plays a direct role in pathways resulting in expression of CXCR5 and Bcl6 (118). Moreover, Tfh from K/BxN mice that develop spontaneous rheumatoid arthritis exhibit a heightened glycolytic demand and inhibition of glycolysis impairs their function reducing disease severity (102). Further support of the requirement of glycolysis for proper Tfh functioning is through reports of human rheumatoid arthritis patients expressing higher levels of hexokinase 2 in peripheral blood mononuclear cells in comparison to their healthy counterparts (119). Moreover, *in vitro* Tfh differentiation is diminished when cultured in low glucose (110). Also, glucose uptake and utilization are enhanced by ICOSL-ICOS stimulation *via* PI3K signaling, and transgenic overexpression of a glucose transporter Glut1 augments Tfh differentiation (110). Together, these data support the claim that Tfh engage a high glycolysis rate; however, the underlying reasons for enhanced glycolytic demand require further investigation. In addition, enhanced glycolytic Tfh dependance has been reported under hypoxic conditions. During malignant pleural effusion (MPE), Tfh cell exhibited differential expression of genes associated with glycolysis, cysteine, and methionine metabolism, while markers of OXPHOS and FAO were slightly elevated. Moreover, these cells show marketed inhibition of the pentose phosphate pathway, arachidonic acid, and α-linoleic acid metabolism (120). Together, this suggests that Tfh may rely on the interconnectedness of various metabolic pathways for fueling cellular demands.

Tfh may also be metabolically regulated by protein kinase C (PKC). Mice that fail to produce PKC-β (*Prkcb^-/-^*) were used to generate a chimera where only B cells were *Prkcb* ^-/-^, while other cell populations were mostly wildtype. These mice exhibit B cells with impaired mitochondrial function and also produce lower numbers of splenic Tfh when compared to wildtype mice, suggesting that B cell expression of PKC-β affects Tfh development (121). Moreover, these mice fail to produce sufficient levels of GCs to elicit an effective immune response, thus they produce lower levels of plasma cells and IgG. These works support the idea that mTOR may regulate mitochondrial respiration through the actions of PKC (121).

Recently, CD4^+^ T cells have been shown to utilize the cytidine diphosphate (CDP)-ethanolamine pathway, the pathway used to produce phosphatidylethanolamine (PE) and plasmenyl PE, a class of phospholipids that form biological membranes (122). The importance of this pathway was related to several intermediates that assist post-transcriptionally in promoting the surface expression and function of CXCR5. The colocalization of CXCR5 and PE at the cell surface is speculated to prevent the internalization and degradation of the critical Tfh homing protein. *In vivo* CRISPR-Cas9 screening identified the CDP-ethanolamine pathway intermediates, as their deletion hindered Tfh differentiation (122). Although this pathway likely does not support Tfh cellular fuel demands for differentiation, it highlights the intricacies of metabolic regulation of Tfh for proper function and suggests that our understanding of these processes is just beginning to scratch the surface.

As there is support for both glycolysis and/or mitochondrial-associated respiration in the regulation of Tfh metabolism, if Tfh can utilize multiple metabolic pathways, it could be conceivably seen as advantageous as it may allow for these cells to adapt to varying conditions within GCs. This may be essential because Tfh cells typically reside within GCs and may frequently have to compete for metabolic resources with other highly proliferative cell types, like B cells. Thus, upon ICOS stimulation Tfh cells can engage glycolysis, but could also maintain function in glycolysis-restricted conditions through utilizing mitochondrial metabolism.

## Tfh in Disease: Autoimmune Related Disorders

Many characteristics of autoimmunity have been closely correlated with an over production of Tfh, including SLE, rheumatoid arthritis (RA), psoriasis, autoimmune thyroid disease (AITD), and diabetes, among others.

Tfh are highly correlated to the disease state in individuals suffering from SLE. It is thought that the major impact of Tfh is due to the disproportionate T:B cell number ratio (123, 124). Recent pre-clinical findings show that long-term glycolytic inhibition *via* 2-DG treatment can normalize frequencies and numbers of autoreactive Tfh, and other hyperactive T and B cells in mice that are predisposed to develop lupus-like symptoms and improve their disease phenotypes (125, 126). This suggests that abnormal glycolysis in these activated cells is involved in lupus progression. However, the mechanisms behind this effect of glycolytic inhibition for the treatment of SLE are still under investigation. Persons suffering from AITD exhibit increased numbers of Tfh in addition to higher levels of Bcl6 and IL-21 mRNA (12, 127). This is in correlation with those afflicted by RA, who exhibit higher concentrations of IL-21 and numbers of circulating Tfh in the bloodstream, likely through enhanced B cell activity (128). Moreover, a link has been established with collagen-induced arthritis (CIA) and the expression of CXCR5. Mice deficient in CXCR5 are resistant to CIA, demonstrating the importance of CXCR5 expression for overall Tfh homing and function, and its potential correlation with CIA (129). Furthermore, it was determined that ICOS signaling in Tfh is linked with activation of PI3K signaling, resulting in expansion of inflammatory T cell populations, and that administration of a glycolysis inhibitor, 3-bromopyruvate, reduced joint inflammation. Together, this suggests a correlation between Tfh ICOS signaling and glycolysis for progression of SLE and rheumatoid arthritis (102, 130).

Tfh have also been implicated in disease severity of other metabolic-associated autoimmune diseases like diabetes and atherosclerosis. The implication of Tfh in these disorders has been attributed to Treg : Tfh imbalance and production of pro-Tfh cytokines, including IL-6 and IL-17A, resulting in increasing disease severity (131, 132). A type 1 diabetes (T1D) mouse model microarray analysis of islet-specific T cells identified an increased gene profile for Tfh, including upregulation of CXCR5 and IL-21 (133). In addition, children that are newly diagnosed with T1D exhibit increased levels of Tfh (134). Moreover, individuals with T1D harbor memory T cells with heightened levels of Tfh markers including CXCR5, Bcl6, and IL-21, and downregulation of IL-2 signaling pathways (133). It is tempting to speculate that the downregulation of IL-2 may be implicated in the elevated levels of Tfh, due to the IL2/Bcl6 axis (133).

CD8^+^ Tfh-like cells have been linked with autoimmune inflammatory diseases in both IL-2 deficient and scurfy-prone mice (85). Scurfy mice are deficient in Treg cells and develop severe autoimmunity due to a lack of Tfh regulation (135). These Tfh-like cells provide B cell help both *in vitro* and *in vivo*, and when in combination with CD4^+^ Tfh (85). This is supported by evidence that depletion of STAT5, specifically in CD8^+^ T cells, resulted in increased numbers of CD8^+^ Tfh-like cells and promotes the production of autoreactive antibodies (136). This is conjuncture with reports linking STAT5 signaling with the negative regulatory role of IL-2 on CD4 Tfh differentiation. CD8^+^ Tfh-like cells have also been shown to contribute to synovial RA. These cells were found within histological sections of GCs and the Tfh-like cells expressed high levels of CD40L, IFN-γ, and lacked expression of perforin in these microenvironments (86, 87). The CD8^+^ Tfh-like cells exhibited IL-17R, are found in circulation and may correlate with prolonged survival (93). Further investigation is warranted to understand the complexities of CD8^+^ Tfh-like and CD4^+^ Tfh mediated responses and how these similar cell populations may synergistically contribute to autoimmunity.

## Tfh in Disease: Cancer

Much of the evidentiary reports of the role of Tfh in cancer are due to studies involving breast cancer and colorectal cancer. Tfh impact multiple aspects of the immune system during cancer. These cells play roles in recruiting natural killer, macrophages, and CD8^+^ T cells, as well as being critical for the formation of ectopic lymphoid structures, all of which are important for an effective anti-tumor response (137). A Tfh gene signature has been described in tumor tissue of breast cancer patients, and expression of this signature correlated with improved tumor clearance and enhanced survival rates and Tfh have been shown to promote B cell differentiation within tumors resulting in improved antitumor immunity (137, 138). For individuals afflicted with colorectal cancer, survival rates have been positively correlated with Tfh numbers. Moreover, tissue microarrays were used to determine that Tfh were more resilient during tumor progression than other T cell populations, the numbers of which experienced progressive reductions (139). Although many sources positively associate Tfh with survival, there are some instances where Tfh are reported to be detrimental to an anti-tumor response, correlated with Tfh production of IL-4. A study using mice lacking CNS2 reported a decrease in tumor growth when compared to wildtype mice. CNS2 is a major source of IL-4 production in Tfh (67). However, in addition to IL-4, Tfh also produce IL-21, which has been shown to promote tumor killing when used in combination with anti-PD-1 or anti-CLTA-4 immunotherapy (140). Although published work for Tfh in the tumor microenvironment is limited, this is an active area of research.

Largely, the metabolic profile of Tfh cells under conditions of cancer is unclear. However, a recent report demonstrates enhanced glycolytic Tfh dependance during malignant pleural effusion (MPE), MPE-derived Tfh cells exhibited differentially enhanced expression of genes associated with glycolysis, cysteine, and methionine metabolism, while markers of OXPHOS and FAO were slightly elevated, when compared to blood-resident Tfh counterparts (120). Moreover, these cells show marketed inhibition of the pentose phosphate pathway, arachidonic acid, and alpha-linoleic acid metabolism (120). Thus, a symphony of multiple metabolic networks is at play under conditions of MPE. The inherently hypoxic environment influences cellular metabolism and has been attributed to increased glycolytic rates and suppression of mitochondrial metabolic pathways (141, 142). From these results we can emphasize the importance of the local environment on cellular metabolism and function. Logically, much of the controversy pertaining to Tfh metabolic reliance can be attributed to these major differences in environmental conditions. Further insight into how Tfh support anti-tumor immunity is undoubtedly forthcoming and will only serve to enhance our understanding of the diverse roles these cells play in cancer, and metabolic cues underlying their differential functionality.

## Tfh in Disease: Infection

The body’s main line of defense against pathogens is through the production of anti-pathogen antibodies. Antibodies are produced by plasma cells that have undergone affinity maturation and expansion within GCs. Tfh are critical for antibody production as they help GC resident B cells differentiate into plasma cells. It has been demonstrated that Tfh play an important role in mounting an immune response against various viruses, bacteria, and parasites, including lymphocytic choriomeningitis virus (LCMV), severe acute respiratory syndrome coronavirus 2 (SARS-CoV-2), human immunodeficiency virus (HIV), *Plasmodium*, *Schistosomiasis*, and influenza infection. Of these, only a few have reported direct evidence between metabolic regulation of Tfh and association with disease. Because of established metabolic links with specific intracellular signaling cascades, some metabolic inferences can be gleaned.

Mice that are infected with a persistent strain of LCMV results in the favorable generation of CD4^+^ Tfh over Th1 cells (143). Due to the relationship between Tfh and B cells to produce antibodies, Tfh are critical for the clearance of viral pathogens. Tfh-mediated clearance of LCMV relies heavily on IL-6 for the increase in Tfh numbers. This increase is linked with the promotion of Bcl6 in these cells (144). Tfh are also critical in mediating a defense against both chronic and acute HCV infection as Tfh numbers increase, identified by expression of CXCR5 and PD-1 (20). Moreover, in humans infected with HCV, Tfh produce more IL-21 and have enhanced expression of ICOS (20), which, given the increase in ICOS, may be associated with enhanced rates of cellular glycolysis (110). These conclusions could be supported by studies in models of murine LCMV infection, where Tfh are described to be long-lived, having persisted for over 400 days. Moreover, these cells were found to be highly glycolytic. These long-lived Tfh, speculated to be memory Tfh, could differentiate into effector and peripheral memory T cell populations (145).

An impaired Tfh and GC response has been found in individuals suffering from COVID-19 caused by SARS-CoV-2 infection. Lung tissue analysis was performed on deceased COVID-19 patients in comparison to healthy individuals who had undergone lung surgery. It was determined that numbers of T and B cells were decreased in patients who had recently died due to COVID-19 (146). Furthermore, reduced lymph node-resident GCs were observed in the deceased patients, correlating with reduced levels of IgG and IgM, when compared to COVID-19 patients that had recovered from disease. Together, the authors speculated that the decreasing GC response and aberrant levels of immune cell populations resulted in a hampered humoral immune response in persons who have died from COVID-19 (146). Increasing expression of ICOS and CXCR5 on CD4^+^ T cells has also been reported (147), and circulating Tfh numbers increase for the duration of infection (148), thus, the increase in ICOS and CXCR5 may be attributed to the enhanced differentiation of Tfh, and increased glycolysis (110). Tfh-ICOS signaling is critical for the generation of an anti-COVID-19 response; hospitalized COVID-19 patients exhibited B cells with a lower expression of ICOS-L, the ligand for ICOS on Tfh, when compared with ambulatory COVID-19 patients (149). Another study has reported that Bcl6 expression in Tfh and B cells decrease, and GCs are significantly reduced during COVID-19 infection (150). Through single cell sequencing another research group has reported that the differentiation of Tfh in COVID-19 patients is impaired and that dysregulated T cell differentiation results in heightened T cell activation and accelerated cell death, with a particular emphasis on CD25^+^ Th1 and Th2 cells (151). Moreover, the authors speculate that altered differentiation may be related to dysregulation of metabolic pathways in these cells, as determined through altered gene profiles, though which metabolic genes are altered, and whether they are glycolytic or OXPHOS genes, is not reported (151). Although Tfh cell numbers have been reported to both increase and decrease, this may be due to differences in disease severity. Meanwhile, another study reported that upon severe COVID-19 infection, a subset of circulating CD4^+^ T cells derived from peripheral blood mononuclear cells (PMBC) express high levels of Voltage-Dependent Anion Channel 1 (VDAC1) and H3K27me3, an epigenetic marker, which may contribute to mitochondrial dysfunction and associated metabolic irregularities (152). Importantly, this distinct T cell subpopulation was not found in individuals with mild/moderate disease, and thus metabolic dysregulation in T cells may contribute to advanced COVID-19 disease (152).

In individuals suffering from HIV, Tfh are the CD4^+^ T cell population with the highest rate of infection *in vivo*, and the single cell type most capable of supporting HIV infection *in vitro* (153, 154). Moreover, HIV-infected Tfh are more likely to be found in GCs (154–156). Upon infection, Tfh do not sufficiently support B cells, though Tfh differentiation is enhanced. The resultant impaired humoral immune response may be due to increased PD-L1 expression on B cells that persistently interacts with PD-1, suppressing the function of Tfh (153). Furthermore, some Tfh exhibit downregulated expression of CD4, PD-1, and CXCR5, and this phenotype is speculated to be a means for infected Tfh to leave the GC and disseminate (154). Although little is known of Tfh-specific metabolic alterations during HIV infection, infected CD4^+^ T cells undergo significant metabolic modulation. HIV manipulates the T cell metabolic environment resulting in a hyperactive and often exhausted T cell phenotype, thereby reducing T cell numbers and overall dysfunction (155, 157, 158). In HIV-infected adults, reduction in T cell function correlates with increases in glucose consumption, due to HIV-induced upregulation of Glut1 in CD4^+^ T cells (159). HIV-infected CD4^+^ T cells exhibit increased amounts of glycolytic intermediates (160) and inhibition of glycolysis reduces the production of viral progeny (161). Moreover, the decline in CD4^+^ T cell numbers could be due to oxidative stress induced by HIV, as HIV-associated proteins can hinder mitochondrial biogenesis (162, 163). Moreover, perhaps HIV is more likely to selectively infect Tfh cells over other CD4^+^ subtypes because of their altered metabolic environment. It appears that viruses can advantageously manipulate the metabolic state of the host cell, to better support infection and resultant production of viral progeny. Addressing these metabolic events underlying pathogen-host interactions will require in-depth single-cell experimentation and analysis.

Parasitic infections are reported to also impact Tfh populations. In mouse models of *Plasmodium* infection, infected IL-6 knockout mice (*Il6^-/-^*) resulted in a more severe infection when compared with infected wildtype mice. Moreover, the authors determined that the expression of IL-6 impacted not only B cell development but also Tfh populations, through increased expression of ICOS as well as enhanced homing of Tfh to splenic B cells (40). Furthermore, investigations in murine schistosomiasis, has shown that Tfh numbers are increased in splenic germinal centers and that the increase in Tfh correlates with progressing liver fibrosis (164). Higher numbers of circulating Tfh have also been demonstrated in human patients infected with *Schistosoma* species when compared with healthy individuals (165). Moreover, it is speculated that IL-4 secreting Tfh may play a role in protection from reinfection by *Schistosoma* species (166). Although direct evidence has not established metabolic alterations with Tfh function in *Plasmodium* infection, over the duration of malaria infection CD4^+^ T cells exhibit an exhausted phenotype (167). Moreover, in a recent report, cellular exhaustion correlated with reduced glycolysis, and was found to be associated with impaired mTORC1 activity (168). Implementation of immunotherapy targeted PD-1, CTLA-4, and IL-27 reduced cellular exhaustion and enhanced cellular rates of glycolysis (168). It is tempting to speculate that this population analysis of CD4^+^ T cells may reflect Tfh metabolic alterations, as mTORC1 has been demonstrated to be required for Tfh differentiation and function (110).

CD8^+^ Tfh-like cells have been described in simian-immunodeficiency virus (SIV) (169). The Tfh-like cells localize to follicles during SIV infection. Interestingly, Tfh-like cell homing to follicles may depend on IL-15, as shown following utilization of human IL-15 superagonist ALT-803. It is speculated that the increased homing of CD8^+^ Tfh-like cells under conditions of the superagonist may correlate with clearance of viral infection, as lower numbers of SIV-infected cells were contained within follicles (169). CD8^+^ Tfh-like cells have also been reported in conditions of chronic LCMV infection. The CD8^+^ Tfh-like cells exhibited many of the hallmarks of CD4^+^ Tfh including CXCR5, PD-1, ICOS, and TFs Batf and Maf, among others (136). Moreover, in Hodgkin’s lymphoma, genetic analysis determined that CXCR5^+^ ICOS^+^ CD8^+^ T cells are most closely related to CD4-derived Tfh than other cell populations (91), and thus these cells may undergo similar metabolic alterations under conditions of infection.

Collectively, Tfh are intimately associated with the severity of numerous diseases. Frequently this is due to dysregulation of these cells resulting in heightened production of pathogenic antibodies. A critical aspect of the manipulation of these cells is likely attributed to modulation of metabolic signaling; yet is a largely unexplored field. Current metabolic findings under conditions of infection are described in **Table 1**. An improved understanding of the metabolic demands of these cells will undoubtedly inform the identification and development of improved therapeutics to treat these diseases.

**Table 1:** Infection-specific metabolic and phenotypic Tfh alterations.

Disease	Tfh-gene alteration affiliated with disease	Tfh disease-associated metabolic pathway	References
HCV	↑ CXCR5, ↑ PD-1, ↑ ICOS	Tfh Unknown	(20)
HIV	↑ CXCR5, ↑ PD-1, ↑ CD4	Tfh-induced OXPHOS prolongs cell survival during infection Glycolysis (CD4^+^ T cells exhibit ↑ glucose consumption, ↑ glycolytic intermediates, ↑ oxidative stress)	(105, 135, 154, 159, 160, 162, 163)
Influenza	↑ ICOS after live -attenuated Influenza vaccination	Glycolysis (2-DG treatment targets autoreactive Tfh)	(126, 170)
LCMV	Moderate ↑ PD-1	Glycolysis (↑ mTOR, ↑ HIF-1, ↑ cAMP, ↑ uptake of 2-NBDG, ↑ baseline ECAR)	(145)
SARS-CoV-2	↓ Bcl6 ↑ ICOS, ↑ CXCR5 (Altered total CD4^+^ T cell population)	Tfh Unknown Glycolysis (Dysfunctional CD4^+^ T cell PBMCs exhibit ↑ VDAC1, ↑ H3K27me3, ↓; Glut1, mitochondrial dysfunction)	(147, 150, 152)
Parasitic infections	IL-6-mediated ICOS expression	Tfh Unknown	(40)

## Conclusions

The importance of Tfh for mounting an adaptive immune response cannot be understated. Tfh are differentiated in response to antigen, and in turn they interact with GC B cells for production of pathogen-specific antibodies. In conditions of good health, Tfh are highly controlled; when this system is disrupted, Tfh may differentiate rapidly, resulting in autoimmunity. Likewise, Tfh differentiation may not occur at rates that allow for a sufficient immune response. In instances of cancer, Tfh may correlate with improved survival rates. However, they require continual access to nutrients to maintain functionality, which in a nutrient-restricted tumor environment often results in an insufficient T cell response for tumor clearance. Under conditions of infection, Tfh are typically associated with the progression of disease. The functionality of these cells is generally altered, resulting in an impaired humoral immune response. As the contributions of CD8^+^ Tfh-like cells to the immune response are now becoming appreciated, experimentation to understand the implications of these cells in generating an immune response under varying disease states will undoubtedly continue. Further investigation and comparison of differing Tfh populations would prove instrumental in unraveling the complexities of Tfh and Tfh-like cell differentiation, and how metabolism may contribute to Tfh development and function.

Arguably, Tfh functionality and differentiation can be largely attributed to metabolic regulation in activated CD4^+^ T cells. Currently, it is unclear whether Tfh ultimately require glycolysis or OXPHOS to fuel metabolic demands, or if these cells can use either, possibly with support of additional metabolic networks, depending on the local environment. It is speculated that many of the differences can be attributed to how Tfh are derived, whether *in vitro* or *in vivo*. Moreover, for *in vivo* implications, how the cells were generated (i.e., by vaccination, infection, or under conditions of autoimmunity, etc.), which may result in differences in metabolic demand according to the methods of activation. Moreover, the tissues-specific microenvironment is undoubtedly a clear metabolic influence, either through induced hypoxia, seen in many diseases, including autoimmunity, viral infections, and cancer. In addition, the competition for resources in these microenvironments are also key contributors to cellular function. These differing conditions could generate the dichotomy in metabolic states. However, currently there appears to be more substantial evidence for the role of glycolysis in fueling Tfh. More work is needed to provide clarity on the metabolic demands of Tfh under differing conditions. This insight will inform the generation of improved, metabolism-targeted, therapeutics for diseases in which Tfh are central, including autoimmunity, cancer, and infection.

## Author Contributions

CM prepared the figures. CM, NL, JW, and C-HC wrote the manuscript. All authors have read and agreed to the published version of the manuscript. All authors contributed to the article and approved the submitted version.

## Conflict of Interest

The authors declare that the research was conducted in the absence of any commercial or financial relationships that could be construed as a potential conflict of interest.

## Publisher’s Note

All claims expressed in this article are solely those of the authors and do not necessarily represent those of their affiliated organizations, or those of the publisher, the editors and the reviewers. Any product that may be evaluated in this article, or claim that may be made by its manufacturer, is not guaranteed or endorsed by the publisher.

## Glossary

2-DG: 2-deoxyglucose
AITD: Autoimmune thyroid disease
AMPK: AMP-activated protein kinase
CIA: Collagen-induced arthritis
cDC: Conventional dendritic cells
COVID-19: Coronavirus disease 2019
CCL: C-C motif chemokine ligand
CDP: cytidine diphosphate
CNS2: conserved non-coding sequence 2
CRCs: CXCL12-producing reticular cells
CXCR: C-X-C motif chemokine receptor
DZ: Dark zone
DC: Dendritic cell
ID: DNA-binding inhibitor
DUSP6: Dual specificity phosphatase 6
ETC: Electron transport chain
LEF-1: Lymphoid enhancer-binding factor-1
Tfh: T follicular helper cell
Tfr: Follicular regulatory T cell
Th: Effector T helper cell
FDCs: Follicular dendritic cells
GC: Germinal center
Glut1: Glucose transport 1
HCV: Hepatitis C virus
HIV: Human immunodeficiency virus
ICOS: Inducible T cell co-stimulator
IL: interleukin
IFN: Interferon
LZ: Light zone
LCMV: Lymphocytic choriomeningitis virus
MHC: Major histocompatibility complex
mTOR: Mammalian target of rapamycin
OXPHOS: Oxidative phosphorylation
Ova: Ovalbumin
PBMC: Peripheral blood mononuclear cell
PD-1: Programmed cell death protein 1
PI3K: Phosphoinositide 3-kinase
PKC: Protein kinase C
SAP: SLAM-associated protein
TCA: Tricarboxylic acid cycle
TCF-1: T cell factor-1
Treg: Regulatory T cell
RA: Rheumatoid arthritis
SARS-CoV-2: Severe acute respiratory syndrome coronavirus 2
STAT: Signal transducers and transcriptional activator
SLAM: Signaling lymphocyte activation molecule
SIM: Simian-immunodeficiency virus
SAP: SLAM-associated protein
SHM: Somatic hyper mutation
SLE: Systemic lupus erythematosus
TCR: T cell receptor
Tfh: T Follicular helper cell
TGF-β: Transforming growth factor-β
TF: Transcription factor
T1D: Type 1 diabetes
VDAC: Voltage Dependent Anion Channel 1
